# EARLY SURGERY IN RARE KNEE HETEROTOPIC OSSIFICATION LEADS TO SUCCESSFUL FUNCTIONAL OUTCOME: A CASE REPORT

**DOI:** 10.2340/jrm-cc.v8.41323

**Published:** 2025-01-03

**Authors:** Stijn PIERREUX, Samar M. HATEM, Stijn ROGGEMAN, Marc SCHILTZ

**Affiliations:** 1 Department of Physical and Rehabilitation Medicine, Universitair Ziekenhuis Brussel; 2 Stimulus Research Group, Vrije Universiteit Brussel; 3 Cluster Neurosciences, Center for Neurosciences (C4N), Vrije Universiteit Brussel; 4 Department of Physical and Rehabilitation Medicine, Faculty of Medicine and pharmacy, Vrije Universiteit Brussel, Brussels, Belgium

**Keywords:** early surgery, functional outcome, heterotopic ossification, knee, rehabilitation

## Abstract

**Background:**

Heterotopic ossification is a common complication after joint replacement surgery, such as hip or knee arthroplasty. In the intensive care unit, it is most commonly associated with traumatic brain injury or spinal cord injury. To prevent recurrence, surgical resection of heterotopic ossification is recommended once the ectopic bone has fully matured, which is estimated to occur after at least 12 months.

**Case presentation:**

This case describes a young woman with no relevant previous medical history who developed severe bilateral heterotopic ossification on the anteromedial sides of her knees after an intensive care unit stay. Passive flexion of both knees was limited to 50°. X-ray was a simple diagnostic tool. Predisposing factors were extended immobilization, prolonged systematic inflammatory condition and mechanical ventilation. Due to the failure of initial conservative therapy, the heterotopic ossification was resected early, 4 months after onset of first symptoms. Following an intensive rehabilitation program, a normal, pain-free gait and full range of motion of both knees were achieved 9 months after surgery.

**Conclusion:**

This case report demonstrates that early resection of heterotopic ossification can result in a good clinical and functional outcome.

## Lay Abstract

Heterotopic ossification is the formation of bone in soft tissues, typically around joints like hip, knee and shoulder, causing significant pain and loss of function in the affected limb. Generally, surgical resection of the excess bone is recommended once the heterotopic ossification is fully matured, which may take at least 12 months. In this case, a young woman developed severe heterotopic ossification on the inner sides of both knees after a prolonged intensive care unit stay. She experienced intense pain, limited knee bending and severely impaired walking. As initial non-surgical treatments failed, the heterotopic ossification was surgically removed just 4 months after the onset of the first pain symptoms. After surgery and an intensive rehabilitation program, she regained pain-free walking and full range of motion in her knees. This case demonstrates that early surgical intervention for heterotopic ossification can lead to good clinical and functional outcomes.

Heterotopic ossification (HO) refers to trabecular bone formation at an abnormal anatomical location, usually in soft tissues around proximal articulations (hips and shoulders). It often leads to significant pain and loss of function in the affected limb. HO is most frequently found after musculoskeletal injuries or after joint replacement surgery, but it also occurs in neurological conditions (spinal cord and traumatic brain injury) and severe burns (1). The incidence of HO after total knee arthroplasty is reported to be around 15% (2). HO after a long intensive care unit (ICU) stay is most commonly associated with traumatic brain injury or spinal cord injury, but is considered a rare complication (3).

Elements of the pathogenesis of HO have been described. In cases of trauma, surgery and burns, an inflammatory environment induces the differentiation of soft tissue muscle-derived mesenchymal stem cells into osteoblastic cells, thus leading to the formation of calcifications or ectopic bone (4). To counter this inflammatory cascade, non-steroidal anti-inflammatory drugs (NSAIDs) are an effective and low-cost treatment in the acute phase or for prevention. Selective and non-selective NSAIDs seem to be equally effective (5).

Stoira et al. (6) have recently described high-risk factors for HO that are associated with ICU stays: extended inflammatory conditions due to the SARS-CoV-2 virus, prolonged immobilization, acute respiratory distress syndrome and mechanical ventilation.

In the ICU setting, similar to other clinical situations, the knee joint is less commonly affected compared to the hip, shoulder and elbow (7). Even though it is unclear from current literature as to when to perform a surgical excision to obtain the best functional outcome at the level of the knee joint, it has generally been suggested that HO should be “cold” for surgery: non-inflammatory and/or not growing. This may take more than 1 year from the start of symptoms (7).

This case report describes the case of a young woman with no relevant previous medical history, presenting with bilateral HO around the knee joint after an ICU stay. The severity of pain and loss of function warranted early excision surgery 4 months after the start of symptoms.

## CASE REPORT

**Fig. 1:**
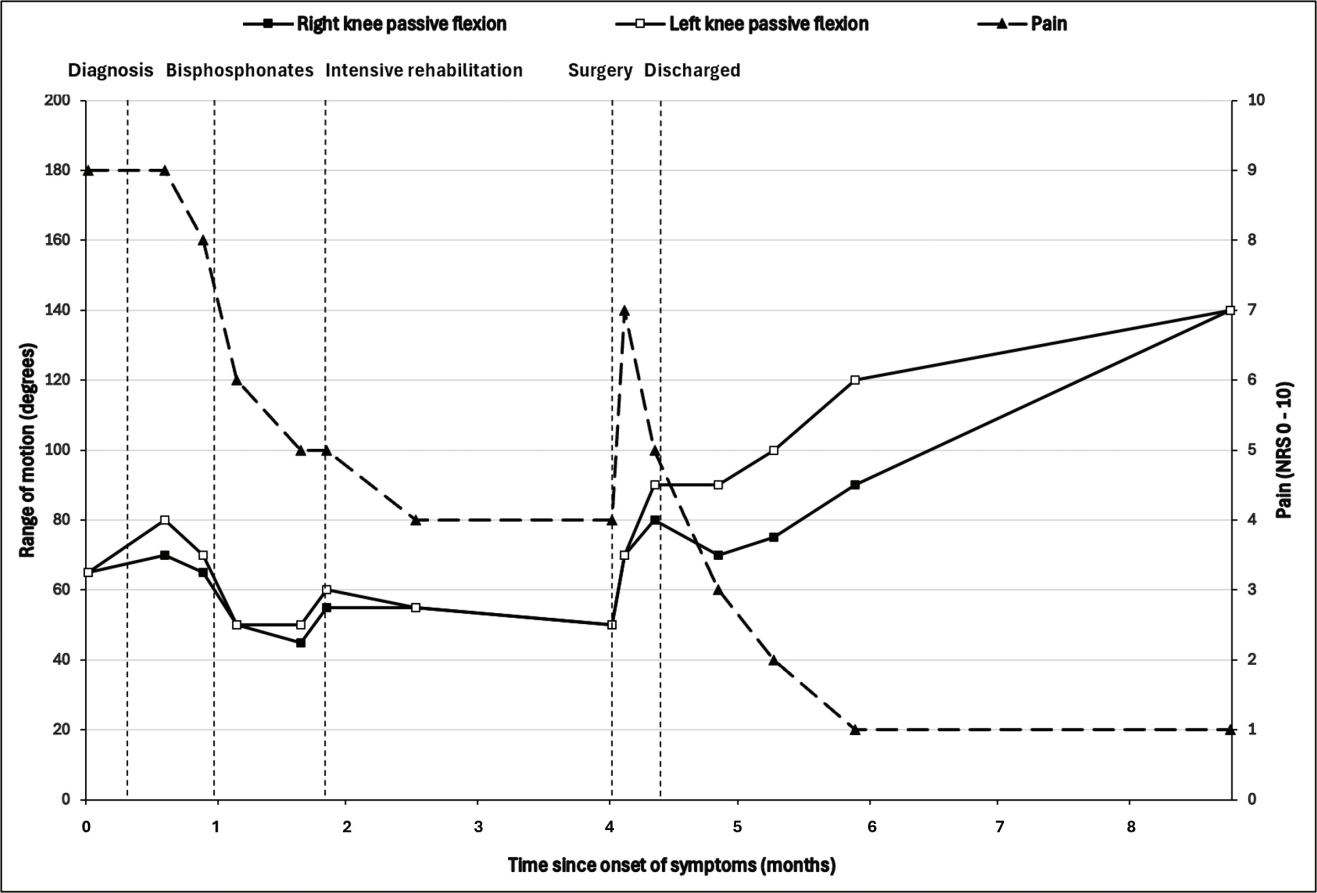
Evolution of the passive range of motion (flexion) of both knees (squares) in degrees assessed with a goniometer, and pain intensity (triangles) assessed with a Numeric Rating Scale ranging from 0 to 10.

A 26-year-old Caucasian woman, 35 weeks pregnant with her first child, was hospitalized for a chorio-amniotic infection. She had no relevant previous medical history and her pregnancy had been uncomplicated until then. After a successful urgent cesarean section, an infected hematoma formed at the site of the incision. Despite a CT-guided drainage, the abscess persisted and a laparotomy was performed to remove it. Subsequently, the patient developed septic shock with acute respiratory distress syndrome. She stayed in the ICU for 28 days, during which she was treated for hospital-acquired pneumonia. Optimal mobilization of the patient during her ICU stay was impeded by medical circumstances: 3 weeks of respiratory intubation, 2 weeks of veno-venous extracorporeal membrane oxygenation with a point of entrance at the groin and respiratory critical illness. Physiotherapy during the ICU stay focused on pulmonary ventilation. After extubation, the patient reported severe pain and stiffness at the anteromedial side of both knees. This pain limited her walking distance to less than 3 m as well as her ability to transfer from bed to chair. Clinical examination of the lower limbs showed a bilateral limitation of passive knee flexion at 65° and active flexion of the knees was almost unobtainable (Fig. 1). Active and passive extension of the knees was preserved. There was a slight effusion of the left knee. Non-depressible masses were palpated on the medial sides of both femoral condyles. Physical therapy was intensified to improve knee mobility. As a result, passive knee flexion marginally improved to 80° (Fig. 1). Persistent pain and limited active knee range of motion had a major impact on patient functioning and mental health. After the ICU stay, the patient was transferred to the infectious diseases department for a duration of 19 days. Subsequently, she was admitted to the inpatient rehabilitation department for gait rehabilitation.

### Diagnostics

**Fig. 2:**
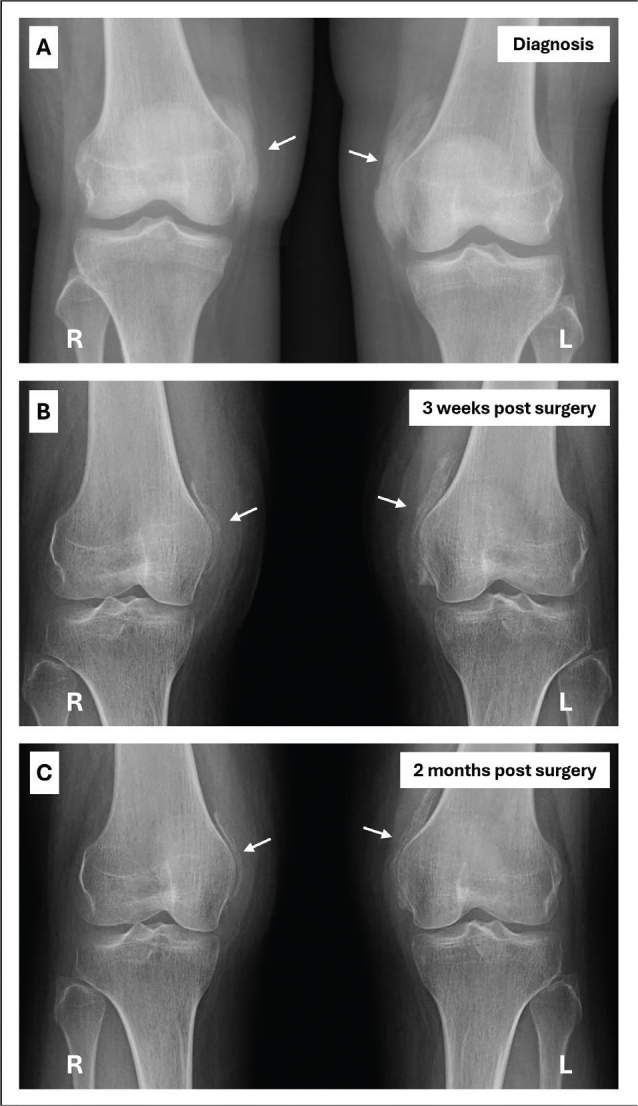
Sequential anteroposterior radiographical images showing bilateral extra-articular calcifications on the anteromedial side of the knees (white arrows). (A) Radiological diagnosis of heterotopic ossification. (B) Three weeks after surgery. (C) Two months after surgery.

**Fig. 3:**
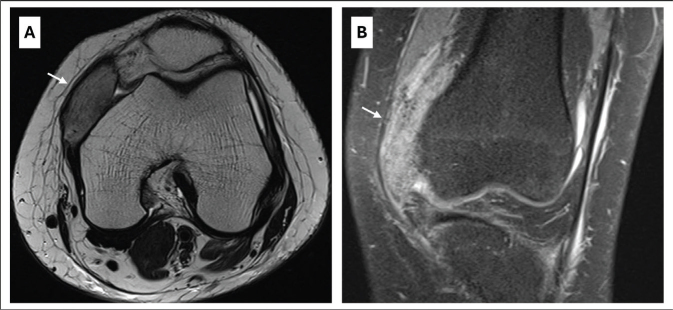
Magnetic resonance imaging of the left knee in a 26-year-old woman without previous relevant medical history, showing extensive heterotopic ossification in the medial femoro-patellar recess with a slight extension to the vastus medius muscle (white arrows). (A) Transversal T2-weighted fast spin echo and (B) Coronal PD-weighted fast spin echo with fat-saturation.

**Fig. 4:**
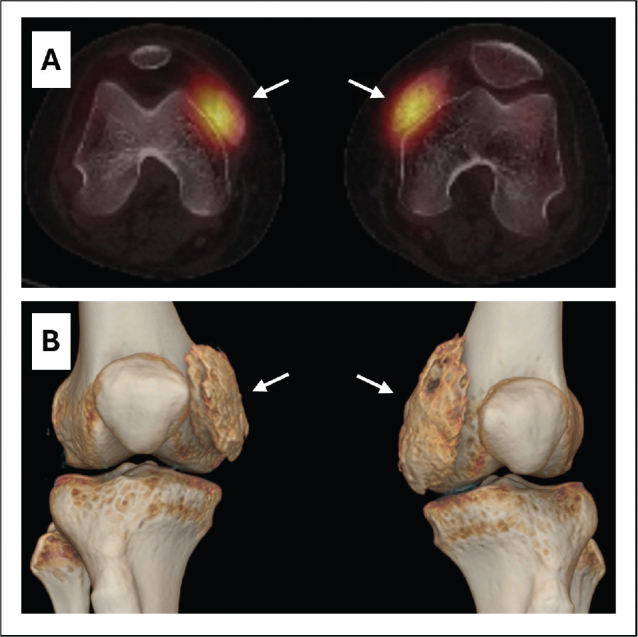
(A) Transversal SPECT-CT of the knees with fused images showing active bilateral heterotopic ossifications 6 weeks after first symptoms. (B) 3D reconstructed image showing the localization of heterotopic ossifications with regard to the knee joint.

**Fig. 5:**
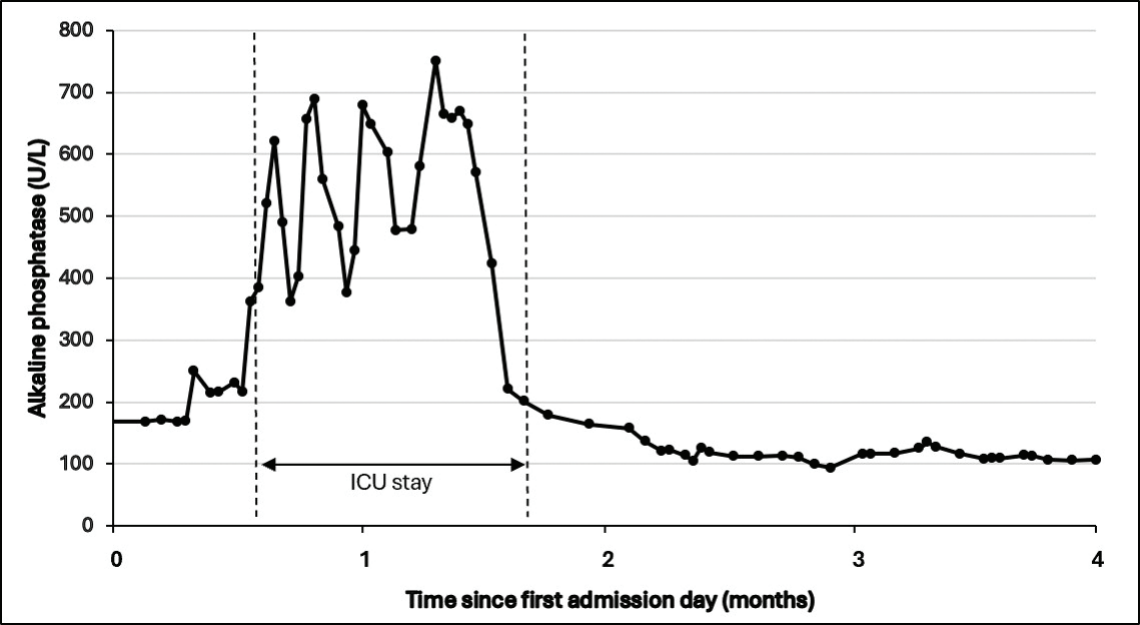
Serum alkaline phosphatase levels during hospitalization showing a peak at 23 days after admission to intensive care unit.

Ultrasonography of the lower limbs excluded deep venous thrombosis and showed a small increase of articular fluid in both knee joints. Standard knee X-rays showed extensive bilateral extra-articular HO on the anteromedial side of both knees without joint abnormalities (Fig. 2A). To better evaluate these HO’s, magnetic resonance imaging (MRI) and single photon emission computed tomography (SPECT-CT) of both knees were performed. MRI showed bilateral HO in the medial femoro-patellar recess with a slight extension to the vastus medialis muscle (Fig. 3). Bone scintigraphy showed actively inflammatory HO’s 6 weeks after the start of symptoms (Fig. 4A). The SPECT-CT images allowed creation of a 3D-modeling image reconstruction, which showed the extent of the HO’s (Fig. 4B). Brain MRI to exclude a central cause for HO, such as stroke or encephalitis, was negative. In retrospect, it was noticed that during the ICU stay, serum alkaline phosphatase (AP) reached a peak at day 23 (751 U/L; normal values 35–104 U/L) (Fig. 5).

### Therapeutic interventions

Initial empirical treatment consisted of intensive physical therapy and patient-controlled analgesia through the intravenous administration of a bolus of 1 mg morphine and 1 mg ketamine if needed. Though considered as a first choice of treatment for HO, non-steroidal anti-inflammatory medication was contraindicated in this patient due to recent major uterine bleeding. A single intravenous perfusion of bisphosphonates (ibandronic acid 3 mg) was administered 56 days after ICU admission. Despite satisfactory pain control and intensive mobilization, the range of motion of both knees decreased to 30° active flexion and 50° passive flexion (Fig. 1). After discussion with the patient and the multidisciplinary team, the decision was made to perform a bilateral surgical resection of the HO. Four months after the onset of symptoms, the patient underwent orthopedic surgery. The HO was detached and resected bilaterally from the medial femoral condyle as completely as possible. The HO appeared fibrous and on the left knee a bony connection had formed with the medial femoral condyle. Following resection, both knees achieved 120° of flexion under general anesthesia. The HO was still partially visible on postoperative X-rays, but showed a significant decrease in density and size (Fig. 2B). Full weight bearing on both legs was encouraged. An intensive in-patient physical therapy program was continued in the rehabilitation department and knee flexion was measured at 90° on both sides 1 week after surgery (Fig. 1). In comparison to pre-operative measurements, 2 months after surgery, the Tinetti Balance Assessment Tool improved from 21/28 to a maximal score of 28/28 and the Functional Independence Measure (FIM) scale increased from 86/126 to 103/126 (8). At the same timepoint and following continuing outpatient physical therapy, passive flexion was measured at 90° on the right knee and 120° on the left knee (Fig. 1) and standard knee radiographs showed stability of the HO (Fig. 2C).

### Final outcome

Thirteen months after the start of the symptoms and 9 months after surgery, both knees reached a normal range of motion of 140° (Fig. 1). No swelling of the knees was observed. Active knee flexion was painless and gait was normal, while palpation of the incisional sites remained slightly painful. To further increase quadriceps strength, out-patient rehabilitation was continued for 8 weeks after which the patient considered herself fully recovered and stopped rehabilitation.

## DISCUSSION

In this case report, severe bilateral HOs were found at the anteromedial side of the knees after an ICU stay in a young woman with no relevant previous medical history. The localization of the HO was unusual and thus led to an uncommon clinical presentation. Plain radiographs proved to be a simple diagnostic tool. Early surgery at only 4 months allowed for a good clinical and functional outcome, and contributed to the prevention of secondary or disuse complications. Thirteen months post-operatively, the knee flexion had increased from 30–50° to 140° bilaterally and she maintained a normal gait without experiencing any pain.

HO may develop during and/or after an ICU stay. To our knowledge, only 2 previous publications have described HO at the medial side of the knee joint after an ICU stay (3, 7). From the reported cases of knee HO, it appears that the anteromedial side of the knee is more likely to be affected than the lateral side (9). The functional impairment seems associated with soft tissue irritation, which can lead to bursitis, impingement and local pain (2).

Early surgery was proposed for this patient after the initial conservative therapy failed to improve the patient’s pain and functional situation. As observed previously, though considered effective as a first-line therapy, NSAIDs were contraindicated for this patient. Previous studies demonstrated that the administration of intravenous bisphosphonates is most efficient in the initial phase when X-rays are still negative (10). In the present case, a single dose of intravenous bisphosphonate, administered after radiographic diagnosis of the HOs, had a moderate effect on pain in the subsequent weeks. Radiation therapy has been shown to be effective in both primary and secondary (after surgery) prevention (1, 10). However, the level of evidence is low (grade 4) and since our patient was still considering future pregnancies, it was decided not to use radiation therapy. According to Shapira et al. (5), prophylactic treatment with NSAIDs remains superior to radiation therapy. The impact of physical therapy on the formation or resolution of HO remains unclear. A comprehensive review reported that either too little or too intensive physical therapy may trigger the formation of HO (1).

As the conservative treatment approach did not provide satisfactory results, the patient agreed to early surgical removal of the HOs. Generally, surgical resection of the HO is recommended after full maturation of the ectopic bone to minimize recurrence, which has been estimated to occur after a minimum of 12 months (7). Mitsionis et al. (7) reported the surgical outcome after the excision of HO around the knee in a series of 14 ICU patients. Knee range of motion, function, and gait improved in a majority of patients with a follow-up of 18 months. However, the latency between the first symptoms and surgery was not reported (7). Lane et al. (3) described a case report with a similar clinical presentation as the present patient, without detailing the therapeutic interventions. HO of the knee has been successfully excised in a reported series of 17 patients with neurological pathologies. The mean interval between the onset of neurological injury and surgical intervention for the HO in this series was 25 months (9).

Extended immobilization, prolonged systematic inflammatory condition due to an infected hematoma, acute respiratory distress syndrome, and mechanical ventilation could have been predisposing factors for the development of HO in our patient. During the ICU stay, serum AP was elevated (>250 U/L). AP is linked with the formation of HO because it suppresses inhibiting factors of bone formation, such as pyrophosphate molecule, which inhibits calcium deposits in non-bony tissues (11). There is, however, no linear relationship between the level of AP in the blood and the severity of HO. AP also can be elevated due to other bone disorders, such as bone metastasis or skeletal trauma, and non-bone disorders such as hepatic dysfunction (11). In the present case, all liver enzymes were elevated due to cholestasis, but AP titer was the highest, making it more likely to be linked with the bone formation process of HO.

This case is of particular interest in that, contrary to the usual timeline for surgical management of HO, surgical resection was proposed and performed at a very early stage. Considering the major functional and emotional impact of the HO, and the insufficient response to multimodal non-surgical therapies with bisphosphonates, analgesics and physical therapy, surgery was performed as early as 4 months after initial symptoms. Though the surgical excision of the calcifications was incomplete and residual bone formations were observed on post-operative radiological imagery, the clinical outcome was still very favorable.
